# Development and Validation of a Rapid Tool to Measure Pragmatic Abilities: The Brief Assessment of Pragmatic Abilities and Cognitive Substrates (APACS Brief)

**DOI:** 10.3390/bs15020107

**Published:** 2025-01-21

**Authors:** Luca Bischetti, Federico Frau, Veronica Pucci, Giulia Agostoni, Chiara Pompei, Veronica Mangiaterra, Chiara Barattieri di San Pietro, Biagio Scalingi, Francesca Dall’Igna, Ninni Mangiaracina, Sara Lago, Sonia Montemurro, Sara Mondini, Marta Bosia, Giorgio Arcara, Valentina Bambini

**Affiliations:** 1 Laboratory of Neurolinguistics and Experimental Pragmatics (NEPLab), Department of Humanities and Life Sciences, University School for Advanced Studies IUSS, Piazza della Vittoria 15, 27100 Pavia, Italy; federico.frau@iusspavia.it (F.F.); chiara.pompei@iusspavia.it (C.P.); veronica.mangiaterra@iusspavia.it (V.M.); chiara.barattieridisanpietro@iusspavia.it (C.B.d.S.P.); biagio.scalingi@iusspavia.it (B.S.); francesca.dalligna@iusspavia.it (F.D.); valentina.bambini@iusspavia.it (V.B.); 2 FI.S.P.P.A. Department, University of Padua, 35131 Padua, Italy; veronica.pucci@phd.unipd.it (V.P.); sonia.montemurro@unipd.it (S.M.); sara.mondini@unipd.it (S.M.); 3 School of Medicine, Vita-Salute San Raffaele University, 20132 Milan, Italy; agostoni.giulia@hsr.it (G.A.); bosia.marta@hsr.it (M.B.); 4 Schizophrenia Research and Clinical Unit, IRCCS San Raffaele Hospital, 20127 Milan, Italy; 5 Department of Psychology, Educational Science and Human Movement (SPPEFF), University of Palermo, 90128 Palermo, Italy; ninni.mangiaracina@community.unipa.it; 6 IRCCS San Camillo Hospital, 30126 Venice, Italy; sara.lago@hsancamillo.it (S.L.); giorgio.arcara@hsancamillo.it (G.A.); 7 University of Padua, 35122 Padua, Italy

**Keywords:** pragmatics, pragmatic impairment, pragmatic disorder, APACS, cognitive-communicative disorder, schizophrenia

## Abstract

Pragmatics is key to communicating effectively, and its assessment in vulnerable populations is of paramount importance. Although tools exist for this purpose, they are often effortful and time-consuming, with complex scoring procedures, which hampers their inclusion in clinical practice. To address these issues, we present the Brief Assessment of Pragmatic Abilities and Cognitive Substrates (APACS Brief), a rapid (10 min), easy-to-use and freely distributed tool for evaluating pragmatics in Italian, inspired by the existing APACS test and already validated in the remote version (APACS Brief Remote). The APACS Brief test measures–with a simplified scale–the domains of discourse production and figurative language understanding and is developed in two parallel forms, each including novel items differing from APACS. Psychometric properties, cut-off scores, and thresholds for change were computed on 287 adults. The analysis revealed satisfactory internal consistency, good test–retest reliability, and strong concurrent and construct validity. Moreover, APACS Brief showed excellent discriminant validity on a sample of 56 patients with schizophrenia, who were also cross-classified consistently by APACS Brief and APACS cut-off values. Overall, APACS Brief is a reliable tool for evaluating pragmatic skills and their breakdown, with brief administration time and simple scoring making it well-suited for screening in at-risk populations.

## 1. Introduction

Pragmatic abilities involve the flexible use of language in different contexts to produce relevant and appropriate discourse and interpret utterances beyond the literal meaning of words (Sperber & Wilson, 2005). While they are crucial for effectively navigating the social world, pragmatic skills are a late achievement during childhood (Domaneschi & Bambini, 2020; Falkum, 2022; Petit et al., 2024; Tonini et al., 2023), reaching full-fledged competence in adulthood and facing a physiological decline in older adults (Jagoe, 2017; Messer, 2015), with possibly severe difficulties in clinical groups (Bischetti et al., 2024a). Diffuse pragmatic breakdowns are documented in psychiatric disorders such as schizophrenia (Agostoni et al., 2024; Bambini et al., 2016a, 2020a, 2025; Colle et al., 2013; Frau et al., 2024a; Parola et al., 2020), as well as in neurological conditions such as amyotrophic lateral sclerosis (Bambini et al., 2016b, 2020b; Bambini & Ceroni, 2021), traumatic brain injury (Arcara et al., 2020; Bosco et al., 2018; Büttner-Kunert et al., 2022), and multiple sclerosis (Carotenuto et al., 2018), among others. Pragmatic assessment was also described as a sensible marker of a broader cognitive decline, even when other cognitive weaknesses are not captured by assessment tools (Lago et al., 2022; Sherman et al., 2021). Furthermore, a fragile pragmatic competence impacts individuals’ quality of life (Agostoni et al., 2021; Snow & Douglas, 2017), highlighting the relevance of pragmatics as a treatment target (Bambini et al., 2020c, 2022).

For the reasons above, assessing pragmatic abilities in vulnerable groups is of paramount importance (Turkstra et al., 2017). In the past decades, the development of assessment tools for pragmatic skills has gained relevance in both research and clinical settings. Assessment tools have been increasingly developed worldwide (Alduais et al., 2023; Cummings, 2018; Verhoeks et al., 2024). Focusing on the Italian context, pragmatic instruments include the Assessment Battery for Communication (ABaCo; Angeleri et al., 2012; Sacco et al., 2008), the Italian version of the *Protocole Montréal d’Évaluation de la Communication* (MEC; Tavano et al., 2013), and the Assessment of Pragmatic Abilities and Cognitive Substrates test (APACS; Arcara & Bambini, 2016). The latter stands out as a comprehensive evaluation of pragmatic skills in the domain of discourse production and figurative language understanding, considering both expressive and receptive aspects. APACS has been used in a vast range of different clinical groups, including both neurological and psychiatric conditions (see Bischetti et al., 2024a; Frau et al., 2024b), and it is available in different languages, such as Hebrew (Fussman & Mashal, 2022), Flemish (Bambini et al., 2021), and French (Petit et al., 2025). As much as other tools, while reliable and precise for research purposes, the duration (lasting approximately 45 min) and scoring procedure of the APACS test make this test often impractical in clinical practice.

In the broader context of neuropsychological assessment, rapid tools are an optimal solution, especially when comprehensive evaluations are not feasible, and short cognitive screenings are preferred for clinical and research purposes (Malloy et al., 1997; Roebuck-Spencer et al., 2017). Rapid assessments are also beneficial for monitoring treatment or disease progression (Snyder et al., 2011). Additionally, patients may struggle with the demands of full assessments, which can be lengthy and resource-intensive (Plass et al., 2010). However, to date, there is a striking dearth of rapid tools for examining pragmatic difficulties (see the exception of the MEC Brief in Brazilian Portuguese by Casarin et al., 2020). The consequence of such a situation is that the evaluation of pragmatic language is rarely incorporated into routine assessment, and communication disorders may often pass unnoticed, increasing the array of underserved populations (Cummings, 2021; Douglas, 2021).

The brief form of the APACS test (APACS Brief) was developed to solve these shortcomings and specifically to allow for a rapid evaluation of communicative skills that considers the main aspects of pragmatic competence. The APACS Brief test is, like APACS, grounded in Gricean pragmatics, where communication is seen as a cooperative activity where speakers need to adhere to rules of appropriateness to context in conducting the verbal exchange and to integrate contextual elements to infer intended meanings (Arcara & Bambini, 2016; Sperber & Wilson, 2005). Discourse production is one domain where the ability to appropriateness to context can be evaluated (Arcara & Bambini, 2016; Prutting & Kittchner, 1987), while specific phenomena that allow testing of pragmatic inferential processes are figurative language expressions, such as metaphors and proverbs, and humor (Attardo, 2024; Carston, 2016). On this basis, the APACS Brief test includes a task evaluating the appropriateness of discourse production and other tasks assessing the ability to derive intended meaning from figurative language and humor. With its rapid (approximately 10 min) administration time, APACS Brief returns a total score that is indicative of both expressive and receptive skills in the domain of pragmatics. Shaped in the latest pandemic scenario, APACS Brief was first validated in its online version, the APACS Brief Remote test, which turned out to be a reliable and valid tool to quickly and flexibly assess pragmatics via videoconferencing on computers and mobile devices (Bischetti et al., 2024b). This work moves ahead in validating APACS Brief for a rapid *in-person* assessment of pragmatic abilities, freely available for clinical and research purposes. Here, we aim to provide its psychometric properties (i.e., reliability and validity), considering a sample of neurotypical individuals as well as individuals with schizophrenia. Moreover, we provide cut-offs and thresholds for significant change across time, following the best practices in the domain of cognitive screening validations (see Aiello et al., 2022).

## 2. Methods

### 2.1. Participants

**Figure 1:**
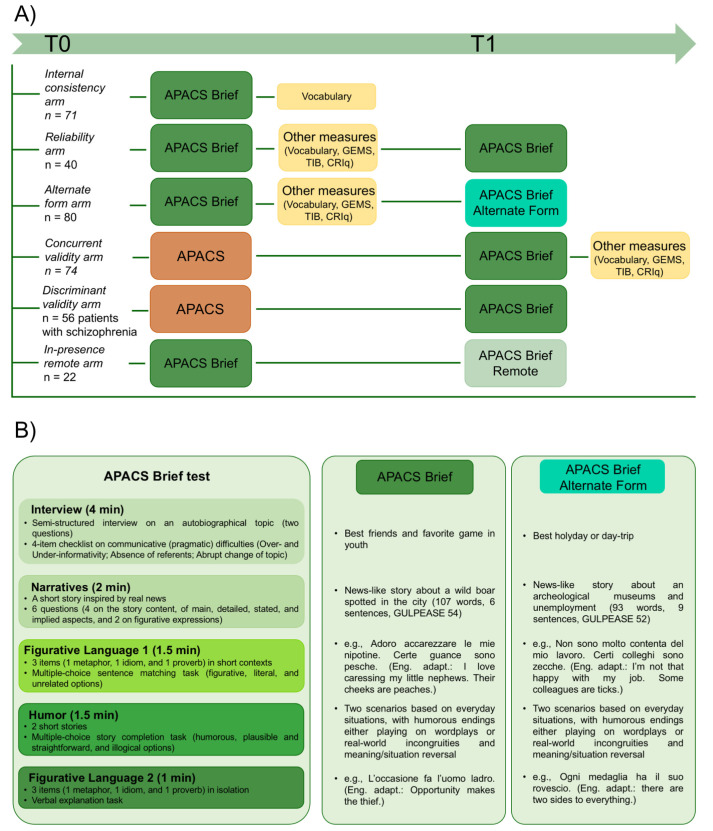
Study design and structure of the APACS Brief test. (**A**) Study design with final samples in each arm. T0 lasted approximately 25 min in the internal consistency arm, 45 min in the reliability, Alternate Form, and concurrent and discriminant validity arms, and 15 min in the in presence-remote arm. T1 lasted approximately 15 min in the reliability, Alternate Form, discriminant validity, and in presence-remote arms, and 40 min in the concurrent validity arm. The assessment included measures of vocabulary (from the Wechsler Adult Intelligence Scale–Revised, WAIS-R; Orsini & Laicardi, 1997), general cognitive (Global Examination of Mental State, GEMS; Mondini et al., 2022), intellectual abilities (Test di Intelligenza Breve, TIB; Colombo et al., 2002), and cognitive reserve (Cognitive Reserve Index questionnaire, CRIq; Nucci et al., 2012). (**B**) Structure of APACS Brief with examples. See the online repository, file 2 (https://osf.io/5xevt/, (accessed on 1 January 2025)) for the psycholinguistic properties of the items of the APACS Brief Alternate Form.

Two hundred and ninety-one adults (180F) participated in the study (Age: M = 43.89, SD = 17.00; Education, i.e., number of attained school/university years: M = 14.00, SD = 3.81). See also Figure 1A. Inclusion criteria were: being a native speaker of Italian; over 18 years of age; no history of neurological, psychiatric, or developmental disorders. A sample of 56 patients with schizophrenia (Age: M = 38.93, SD = 12.91; Education: M = 11.98, SD = 2.75; 7F)—diagnosed following the DSM-5 criteria—were also included in the study to evaluate discriminant validity of APACS Brief and comparison with the APACS test (Arcara & Bambini, 2016).

The study was approved by the Ethics Committee of the Department of Brain and Behavioral Sciences of the University of Pavia (n. 041/2020, granted on 18 February 2020), by the Ethics Committee of Psychological Research (Area 17) of the University of Padua (n. D0D54514D98A549EC1C48C082DA4987C, granted on 29 April 2020), and by the Ethics Committee of the IRCCS San Raffaele Hospital, Milan (n. 234/2019, granted on 25 January 2021).

### 2.2. Design and Assessment

The study was designed to include multiple arms, that is, distinct groups of participants assigned to different testing sessions. Each arm was used to evaluate relevant aspects of the APACS Brief test. The design included two sessions (T0 and T1), scheduled to take place approximately two weeks apart (±four days). All participants were administered APACS Brief at the first (T0) or second session (T1), or at both time-points (see Figure 1A). Together with the first (or single) administration of APACS Brief, they also completed further linguistic, pragmatic, or cognitive tests, depending on the arm to which they were assigned (see Figure 1A). At the end of the first APACS Brief administration, participants were invited to complete a short debriefing session. Data were collected by trained and in-training psychologists and linguists. An independent rater re-scored part of the data to assess inter-rater reliability, adopting the scoring manual provided with the test.

#### 2.2.1. APACS Brief—Test Construction

The APACS Brief test is a rapid assessment tool measuring expressive and receptive pragmatic skills in the domains of discourse and non-literal language. It was developed following the theoretical and structural features of the APACS test, with modifications aiming at reducing administration time and scoring complexity. Compared to APACS, the number of items in APACS Brief was reduced by 85%, for a total administration time of ~10 min, yet including the same tasks (except for Description). In particular, APACS Brief includes five tasks (see Figure 1B):

- Interview. A semi-structured interview assessing conversational abilities through two autobiographical topics (i.e., best friends and favorite games in youth), focusing on over- and under-informativeness and discourse flow. Max score: 4;
- Narratives. Participants listen to a short story (i.e., a radio news about a wild boar in the city) and are asked to answer comprehension questions about both stated and implied aspects, including questions on figurative language (e.g., explaining the meaning of an idiom contained in the story, “take the bull by the horns”). The news-like story comprises 107 words across six sentences. With a Gulpease readability index score of 54 (Lucisano & Piemontese, 1988), the text is moderately challenging for readers with an educational background equivalent to eight years of schooling. Max score: 6;
- Figurative Language 1. Participants are asked to select the correct figurative interpretation of three non-literal expressions presented in short contexts (e.g., Italian, “Adoro accarezzare le mie nipotine. Certe guance sono pesche”; English translation, “I love caressing my little nephews. Their cheeks are peaches”), choosing between three options, one correct (Italian, “Certe guance sono lisce e morbide”; English translation, “Some cheeks are smooth and soft”), one literal (Italian, “Certe guance sanno di frutta”; English translation, “Some cheeks taste peachy”), and one unrelated (Italian, “Certe guance sono rugose”; English translation, “Some cheeks are wrinkled”). Items include one idiom (with a familiarity rating of 6.29 and a literal plausibility of 6.6 on a 7-point scale, as per Tabossi et al., 2011), one metaphor (with a familiarity rating of 2.9 on a 5-point scale, according to Bambini et al., 2013), and one well-known proverb (documented in the itWaC corpus, based on Lambertini, 2016, 2022). Max score: 3;
- Humor. Participants are asked to choose the punchline of two stories (e.g., Italian, “In un hotel in montagna un cliente si lamenta con l’albergatore: ‘Vuole duecento euro per la stanza? Ma quest’estate mi ha fatto pagare solo cento euro!’ E l’albergatore:”; English translation, “At a mountain hotel, a guest complains to the hotel owner: ‘You want 200 euros for the room? But this summer you only charged me 100 euros!’ The hotel owner replies:”) from three options, one unexpected and humorous (Italian, “Beh, qui in montagna d’inverno le notti sono più lunghe”; English translation, “Well, here in the mountains, winter nights are longer”), one literal and coherent (Italian, “Capirà, l’inverno è alta stagione”; English translation, “Well, winter is high season”), and one non-relevant and absurd (Italian, “Abbiamo un ampio parcheggio gratuito.”; English translation, “We offer a spacious free parking lot”). Items were drawn from previous works on the pragmatics of humor and are moderately witty (with a mean funniness of 3.89 on a 7-point scale, based on Canal et al., 2019). Max score: 2.
- Figurative Language 2. Participants are asked to produce verbal explanations of figurative expressions presented in isolation (e.g., Italian, “L’occasione fa l’uomo ladro”; English translation “Opportunity makes the thief”). Items include one metaphor (with a familiarity rating of 3.33 on a 5-point scale, according to Bambini et al., 2013) and two proverbs (documented in the itWaC corpus, based on Lambertini, 2016, 2022). Max score: 3.

Importantly, for APACS Brief, new items were created to match the structure and psycholinguistic properties of the APACS items that showed the highest correlations with the APACS total scores (as described in Bischetti et al., 2024b). Furthermore, we checked that none of the new items was included in other tools often used in combination with the APACS and possibly APACS Brief, namely the Positive and Negative Syndrome Scale (PANSS; Kay et al., 1987), the Wechsler Adult Intelligence Scale–Revised (WAIS-R; Wechsler, 1981), and the Pragmatics of Communication (PragmaCom) training program (Bambini et al., 2022, 2020c). For the Interview task, novel autobiographical topics compared to APACS were selected, inspired by previous work (Ruffman et al., 2010). Finally, the scoring procedure was simplified from a 3-point (incorrect/0; partially correct/1; correct/2) to a binary scale (incorrect/0; correct/1).

Tasks are administered orally, supported by an additional booklet showing the response options for the Figurative Language 1 and Humor tasks, in a fixed order. Task scores are proportionally transformed and then averaged, thus ensuring that each task equally contributes to the total score, which ranges from 0 to 1 (similar to the approach used for the APACS test, Arcara & Bambini, 2016, and cognitive measures, e.g., the Global Examination of Mental State, GEMS; Mondini et al., 2022). Importantly, APACS Brief is equipped with a detailed manual for raters, which includes examples based on data collected during pilot sessions, and further updated when needed.

The APACS Brief test was designed together with an alternate version (henceforth APACS Brief Alternate Form), to use for retesting and monitoring over time while reducing potential practice effects. The APACS Brief Alternate Form included another set of novel items (i.e., different from the ones included in the APACS Brief test as well as from APACS ones) with comparable psycholinguistic properties. As such, the two versions were expected to correlate at least moderately and to be statistically equivalent.

#### 2.2.2. Further Pragmatic and Cognitive Assessment

According to the study arm to which they were allocated, participants were also assessed for pragmatics via other tools and for further linguistic and cognitive skills. The pragmatic assessment included the extended APACS test (Arcara & Bambini, 2016), a validated tool assessing communicative skills including six tasks in the production (i.e., the Interview and Description tasks) and comprehension domains (i.e., the Narratives, multiple-choice Figurative Language, Humor, and verbal-explanation Figurative Language tasks), which returns a Total APACS score (range 0–1), and the APACS Brief Remote, namely, the APACS Brief test administered via teleconferencing.

Other participants were assessed for vocabulary skills, general cognitive profile, intellectual abilities, and cognitive reserve. Vocabulary was assessed via the vocabulary subtest of the Wechsler Adult Intelligence Scale–Revised (WAIS-R; Wechsler, 1981), in Italian (Orsini & Laicardi, 1997): the task is composed of a list of 35 words of increasing difficulty, and participants are asked to verbally explain the meaning of each word. The general cognitive profile was evaluated with the Global Examination of Mental State (GEMS; Mondini et al., 2022). The GEMS is a screening test composed of 11 items assessing different cognitive domains (e.g., spatial navigation, memory, and shifting). Furthermore, intellectual abilities were measured with the analogue of the National Adult Reading Task in Italian (Test di Intelligenza Breve, TIB; Colombo et al., 2002). The TIB consists of a rapid reading of 54 Italian words, 20 with high and 34 with low lexical frequency, the latter containing both regularly and irregularly stressed words. It returns an (adjusted) score for verbal, non-verbal, and general intelligence. Finally, Cognitive reserve (CR) was measured with the Cognitive Reserve Index questionnaire in Italian (CRIq; Nucci et al., 2012). The CRIq consists of a semi-structured interview capturing sources of CR in the lifespan, from education (CR-Education) to occupation/working undertakings (CR-Work) and leisure/free-time activities (CR-Leisure).

### 2.3. Statistical Analysis

Internal consistency was determined by calculating single items’ scores, the (polychoric) Cronbach’s alpha, and task-total correlations.

Inter-rater reliability was calculated via two-way average agreement Interclass Correlation Coefficients (*ICC*) of total and task scores.

Test–retest reliability was verified by correlating T0 and T1 scores, and practice effects with *t*-tests.

The equivalence between forms was calculated via a correlation analysis (testing the significance for a difference with the test–retest coefficient using the Fisher *r*-to-*z* transformation) and a multi-step form equivalence testing procedure (along the lines of Bischetti et al., 2024b). Performances in the two forms were examined by the comparison of: summary scores (means, SDs, and mean difference); total scores via dependent-sample *t*-tests and Two One-Sided Tests (TOST) *t*-tests of equivalence (Lakens et al., 2018), with equivalence test bounds based on the smallest effect size of interest (SESOI) of *d* = 0.32, determined with a two-tail sensitivity analysis with 80% power, a sample size of 79, and α = 0.05; Standard Errors of Measurement (SEM) against the mean difference between total scores; and total scores at the individual level with a Bland-Altman analysis of agreement (Bland & Altman, 1986).

The effects of demographic variables (i.e., age, education, and sex) were examined with correlational and (linear and orthogonal second-order polynomial) regression analyses, the latter scrutinized by jointly evaluating the results of Likelihood Ratio Tests (LRTs) and weighted second-order Akaike Information Criteria (AICc) values. Cut-off scores and significant change thresholds for total scores were derived following a regression-based method (Crawford & Garthwaite, 2006) accounting for relevant demographic predictors.

A discriminant analysis on APACS Brief total scores between individuals with schizophrenia and neurotypical adults was performed with a ROC analysis. Furthermore, the association between the APACS Brief and APACS tests (i.e., concurrent validity) was studied via a correlation (on the sample of patients with schizophrenia and neurotypical adults who underwent the APACS Brief and APACS assessment; see also Arcara et al., 2019) and a cross-classification analysis of above and below cut-off performance in both tests (on the sample of patients with schizophrenia) using a contingency table and a chi-square test (with Yates’ correction). Construct validity was assessed via correlations with the cognitive assessment in samples of healthy adults. Finally, the equivalence between testing modalities was assessed with a correlation analysis.

Summary data are presented along with their 95% confidence intervals (CIs).

Analyses were performed with R, v. 3.6.2 (R Core Team, 2022).

## 3. Results

### 3.1. Sample Characteristics

A total of 287 adults (180F) completed at least one experimental session with the APACS Brief (drop-outs, n = 4). The final sample is described in Table 1.

### 3.2. Internal Consistency

Internal consistency, calculated on the whole sample of participants assessed with the APACS Brief (from the internal consistency arm and all data points from the other arms at T0 or T1), showed an acceptable Cronbach alpha value (*α =* 0.73, CI [0.68, 0.77]). All tasks showed moderate-to-strong correlations with the total score: Interview–Total, *r* = 0.32, CI [0.21, 0.42], *p* < 0.001; Narratives–Total, *r* = 0.54, CI [0.46, 0.62], *p* < 0.001; Figurative Language 1–Total, *r* = 0.48, CI [0.39, 0.57], *p* < 0.001; Humor–Total, *r* = 0.58, CI [0.50, 0.65], *p* < 0.001; Figurative Language 2–Total, *r* = 0.74, CI [0.69, 0.79], *p* < 0.001. Overall, APACS Brief showed good internal consistency.

### 3.3. Inter-Rater Reliability

Inter-rater reliability, assessed on the scores of 80 participants (27.88%), revealed an excellent agreement for the total (*ICC* = 0.97, CI [0.95, 0.98], *p* < 0.001) and good agreement for task scores (*ICC*s ≥ 0.87, *p* < 0.001). Overall, the APACS Brief test showed excellent inter-rater reliability.

### 3.4. Test–Retest Reliability and Practice Effect

Test–retest reliability, measured on the 40 participants in the reliability arm (Age: M = 47.63 years, SD = 19.19; Education: M = 13.63 years, SD = 3.96; 23F), revealed a strong association between performances over time (*r* = 0.66, CI [0.44, 0.81], *p* < 0.001). A small practice effect emerged (Δ_total scores_ = −0.04, *t*(39) = −2.16, *p* = 0.037). See the online repository, file 1 (https://osf.io/auhgm/, (accessed on 1 January 2025)) for further details. Overall, the APACS Brief test showed good test–retest reliability and a limited practice effect.

### 3.5. Alternate Form Reliability and Equivalence

The analysis of the Alternate Form, conducted on the participants in the Alternate Form arm with one exclusion (n = 79; Age: M = 45.79 years, SD = 20.69; Education: M = 14.67 years, SD = 3.92; 43F), showed that it is as reliable as the main form (*α* = 0.79, CI [0.72, 0.85]). The correlation between forms was moderate (*r* = 0.48, CI [0.29, 0.63], *p* < 0.001), and testing the difference between the correlations of APACS Brief scores at T0 and T1 (test–retest) and the correlation of APACS Brief and its Alternate Form yielded a non-significant result (Δ_r_ = 0.18, CI [–0.09, 0.42], *z* = 1.36, *p* = 0.173). Total scores for the two forms were similar as *per* performance means and SDs (0.83 ± 0.12 and 0.84 ± 0.13), with a negligible mean difference (Δ_Mean_ = −0.01) and similar SEM (0.01 and 0.02). Total scores were not significantly different (*t*(78) = −0.49, *p* = 0.627) and they were significantly equal (*t*(78) = 21.92, *p* < 0.001). Based on the null hypothesis test and the equivalence test combined, we can conclude that APACS Brief and its Alternate Form are moderately related and not significantly different from one another. Differences at the individual level with the Bland-Altman analysis showed a distribution around zero, with only three points (that is, three participants representing 3.8% of this sample) falling beyond the upper and lower limits of agreement, thus indicating very limited distortion. For the complete set of results, see the online repository, file 2 (https://osf.io/5xevt/, (accessed on 1 January 2025)). Overall, the APACS Brief and its Alternate Form can be intended as equivalent.

### 3.6. Effects of Demographics

Total scores were negatively correlated with age both for the main and the alternate versions (APACS Brief: *r* = −0.31, CI [−0.41, −0.20], *p* < 0.001; Alternate Form: *r* = −0.50, CI [−0.65, −0.32], *p* < 0.001) and positively with years of schooling (APACS Brief: *r* = 0.35, CI [0.25, 0.45], *p* < 0.001; Alternate Form: *r* = 0.55, CI [0.38, 0.69], *p* < 0.001).^1^ No effect of sex was found (APACS Brief: *r* = −0.02, CI [−0.14, 0.09], *p* = 0.679; Alternate Form: *r* = −0.18, CI [−0.39, 0.04], *p* = 0.106).

**Figure 2:**
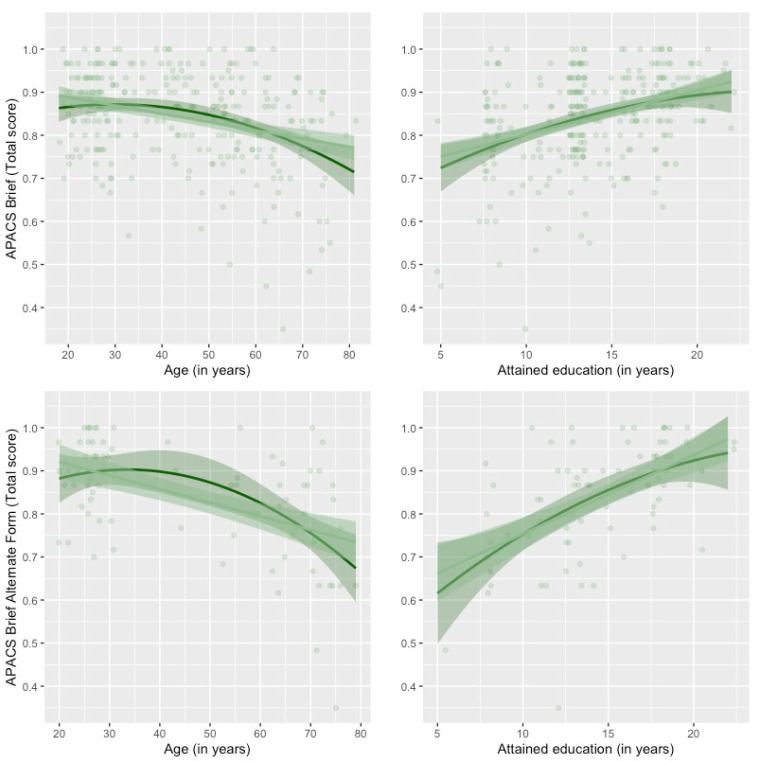
Visual representation of the regression coefficients for the role of demographic variables in APACS Brief and its Alternate Form total scores. The light green line corresponds to the linear term and the dark green line to the second-order polynomial term introduced in the regression analysis, plotted with their color-matching 95% confidence intervals. A position adjustment (jitter) for the observations was added for visualization purposes.

In the regression analysis, age exerted a significant linear (APACS Brief: *β* = −0.38, *t* = −3.57, *p* < 0.001; Alternate Form: *β* = −0.29, *t* = −2.32, *p* = 0.023) and quadratic effect (APACS Brief: *β* = −0.20, *t* = −1.96, *p* = 0.051; Alternate Form: *β* = −0.23, *t* = −2.08, *p* = 0.041), whereas education had a linear effect only (APACS Brief: *β* = 0.49, *t* = 4.65, *p* < 0.001; Alternate Form: *β* = 0.46, *t* = 3.74, *p* < 0.001). No effects of the quadratic term of education (*p*s > 0.147) and sex emerged (*p* > 0.136). Results are plotted in Figure 2.

### 3.7. Cut-Offs and Significant Change Thresholds

In the regression analysis, the models that best explained total scores’ variance included the linear terms of age and education (APACS Brief: 49.1%, AICc(4) = −503.38; Alternate Form: 31.6%, AICc(4) = −126.74). No clear polynomial effects (as indicated by LRTs) and no (general) role of sex emerged; hence, cut-offs were not split for sex, and polynomial terms were not considered.

Cut-offs were then computed on the basis of the linear terms of age and education: values are available in the online repository, file 3 (https://osf.io/dfcjg/, (accessed on 1 January 2025)), along with significant change thresholds.

### 3.8. Discriminant Validity

**Figure 3:**
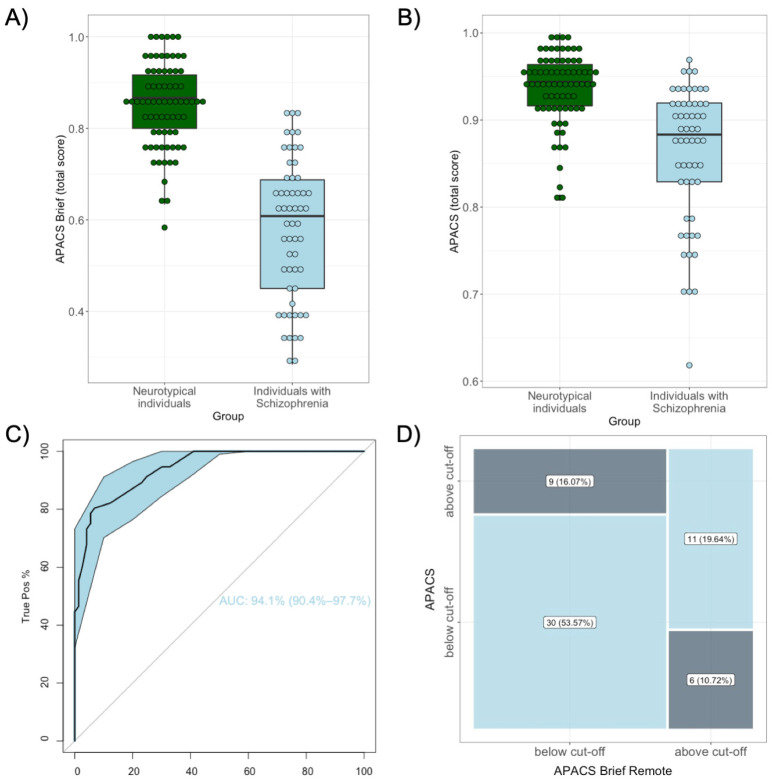
Discriminant validity and APACS-APACS Brief cross-classification analysis. (**A**,**B**) Comparison of mean scores obtained in the APACS Brief and APACS tests individuals with schizophrenia (in light blue) and between neurotypical individuals (in green, n = 73, coming from the concurrent validity arm), with independent samples *t*-tests *p*-values; (**C**) ROC analysis discriminating between patients and controls, with AUC value; (**D**) Cross-classification of performance above and below normative cut-off in the APACS and APACS Brief tests in the sample of patients with schizophrenia.

The sample of patients with schizophrenia (n = 56) obtained an average APACS Brief total score of M = 0.58 (SD = 0.15). Compared to the sample of neurotypical individuals from the concurrent validity arm with one exclusion (n = 73), patients with schizophrenia scored significantly lower in the APACS Brief test (*t*(85.90) = 11.76, *p* < 0.001; Figure 3A). The AUC of the ROC model discriminating between these samples was 0.94 (CI [0.91, 0.98]; specificity = 93.15%; sensitivity = 80.36%, Figure 3C), showing an excellent discriminant validity, with an optimal point (Youden index) at 0.725.

### 3.9. Concurrent Validity, Cross-Classification Analysis, and Construct Validity

The association between the APACS Brief and APACS tests (concurrent validity) was assessed by combining the sample of patients with schizophrenia and the sample of neurotypical adults from the concurrent validity arm with one exclusion. The correlation between total scores was strong (*r* = 0.71, CI [0.61, 0.79], *p* < 0.001). Moreover, patients with schizophrenia (M = 0.86; SD = 0.08) scored lower than neurotypical adults also in the APACS test (*t*(78.31) = 6.49, *p* < 0.001; Figure 3B).

The cross-classification analysis in the clinical sample showed a stable pattern of co-occurrence between scores above or below the normative cut-off in APACS Brief and APACS (*χ*^2^(1) = 7.22, *p* = 0.007, Figure 3D), with 73.21% of patients correctly classified by APACS Brief. In particular, 53.57% of patients scored below and 19.64% above the normative cut-off in both tests. Only 26.79% of subjects were categorized differently by APACS Brief compared to APACS, 16.07% as false positive and 10.71% as false negative. Overall, the model’s accuracy was 73.21% (CI [59.70, 84.17]): APACS Brief has fair specificity (64.71%) and good sensitivity (76.92%) in matching the classification of the more comprehensive APACS test.

As per the construct validity, carried out on the samples specified in the note of Table 1, the APACS Brief total score moderately correlated with the WAIS-R vocabulary subtest (*r* = 0.45, CI [0.30, 0.57], *p* < 0.001) and the GEMS (*r* = 0.50, CI [0.37, 0.61], *p* < 0.001), and weakly with verbal (*r* = 0.35, CI [0.21, 0.47], *p* < 0.001) and general intellectual abilities (*r* = 0.26, CI [0.11, 0.39], *p* = 0.001). Furthermore, a series of other interesting—albeit very weak—correlations were found, specifically with performance IQ (*r* = 0.14, CI [−0.02, 0.28], *p* = 0.080) and the schooling index of the CRI (*r* = 0.17, CI [0.01, 0.32], *p* = 0.037). For the complete set of correlations, see the online repository, file 1 (https://osf.io/auhgm, (accessed on 1 January 2025)). Overall, results highlighted a meaningful pattern of associations between the APACS Brief and the general cognitive substrate.

### 3.10. Equivalence of the APACS Brief in Presence and Remote Version

On the sample of participants in the in presence-remote arm with one exclusion (n = 21), performance between the in-person and remote assessment was strongly correlated (*r* = 0.89, CI [0.74, 0.95], *p* < 0.001). The experience between modalities, evaluated with a bipolar semantic differential scale (in-person, −1/−3; no difference: 0; online: +1/+3), revealed no difference in terms of difficulty (M = 0.20, SD = 1.08; *t*(14) = 0.72, *p* = 0.486) and involvement (M = −0.73, SD = 1.41; *t*(14) = −1.85, *p* = 0.085). However, participants were more satisfied (M = −0.87, SD = 1.53; *t*(14) = −2.39, *p* = 0.032) and overall preferred the in-person session (M = −1.13, SD = 1.64; *t*(14) = −2.67, *p* = 0.018). No effect of demographic features was found (*p*s ≥ 0.073). Overall, APACS Brief produces very similar results in the two testing modalities.

### 3.11. Debriefing

After completing APACS Brief, participants positively judged how tasks and questions were presented, described themselves as motivated in performing the task, and reported no fatigue (but a single participant). The Interview, Narratives, and Figurative Language 2 tasks were evaluated as the most challenging ones. The vast majority of participants were satisfied with their performance and some overtly expressed their appreciation for the short time it took to complete. Overall, the APACS Brief is rated as not difficult and satisfactory, with minor challenges associated with some tasks.

## 4. Discussion

In this work, we presented the APACS Brief test, a tool designed to offer a rapid assessment of pragmatic abilities, along with its psychometric properties and cut-off scores based on a well-represented sample of the Italian population. The extensive study of its properties convincingly demonstrates that the APACS Brief is a reliable tool with satisfactory psychometric properties, similar to those obtained for its remote version (Bischetti et al., 2024b) and for other yet longer pragmatic tests, such as APACS (Arcara & Bambini, 2016) or ABaCo (Angeleri et al., 2012; Bosco et al., 2012; Sacco et al., 2008). Hence, APACS Brief can be considered a useful tool to assess pragmatics in all those contexts that pose time constraints, to reduce the burden of full-length assessments, or to monitor treatment efficacy and disease progression over time.

In particular, the internal consistency of APACS Brief is acceptable (*α* = 0.73) and in line with values previously found for APACS Brief Remote (*α* = 0.69; Bischetti et al., 2024b) and APACS (0.60 < *α* < 0.70; Arcara & Bambini, 2016). APACS Brief is also stable over time (*r* = 0.66), as previously shown for its online version (*r* = 0.69; Bischetti et al., 2024b). The inter-rater reliability of APACS Brief indicated satisfactory concordance (*ICC*s ≥ 0.87), in line with values reported for the APACS total scores (0.57 < *ICC*s < 1; Bambini et al., 2016a).

The validity of APACS Brief is supported by the robust correlation with the more fine-grained APACS test (*r* = 0.71), as well as its moderate correlations with linguistic and general cognitive and intellectual abilities. Concurrent and construct validity are accompanied also by excellent discriminant validity, denoting the ability of APACS Brief to single out neurotypical adults from psychiatric patients based on pragmatic skills, in line with the literature describing the marked pragmatic deficit of individuals with schizophrenia (e.g., Bambini et al., 2016a, 2020a). While the correlation between APACS Brief and APACS does not reach the level observed for other short tests compared to their longer forms (e.g., for the short vs. the standard versions of the MoCA test, *r* = 0.96; Roalf et al., 2016, or for the Boston Naming Test, *r* = 0.94; Grima & Franklin, 2016), it is important to recall that, differing from other brief tools, APACS Brief includes novel items not derived from APACS. In this view, the magnitude of the association between APACS Brief and APACS outperforms the one between the ex-novo brief and the extended Brazilian MEC (*r*s < 0.62; Casarin et al., 2020) and aligns with those of novel short language tests compared to established measures, such as the Mini Linguistic State Examination against the Boston Diagnostic Aphasia Examination and the Assessment of Comprehension and Expression (between *r* = 0.60 and *r =* 0.67; Patel et al., 2022) and the Brief Executive Language Screen against the Cookie Theft scene description (*r* = 0.74; Robinson et al., 2022). Moreover, the concurrent validity measured against APACS improved remarkably from the remote to the in-person version of APACS Brief, with a notably stronger association (*r* = 0.38 vs. *r* = 0.71). Two main differences can be considered: first, APACS Brief and APACS were here administered in the same modality, i.e., in similar in-presence, non-pandemic, and possibly more relaxed contexts; second, this study involved a greater sample size (n = 129 vs. n = 28), which also included a wider range of demographic background (especially in terms of schooling, M = 13.57 ± 3.79 vs. M = 16.43 ± 3.39) and clinical background (comprising patients with schizophrenia), thus resulting in a more stable (and greater in magnitude) correlation coefficient.

The most important aspect of the validation of APACS Brief is perhaps its ability to capture the presence of a pragmatic disorder in clinical groups, which appears overall comparable to that of the longer APACS, here taken as the gold standard. The cross-classification analysis conducted on the group with schizophrenia evidenced that the vast majority (73.21%) of patients were similarly classified as above or below the normative cut-offs by APACS Brief and APACS. APACS Brief was proved to have good clinical sensitivity against APACS in detecting pragmatic difficulties, but a relatively lower specificity, as a number of false positives emerged (16.07%). This needs to be taken into account when deciding whether to use this brief, rather than the full version, making the APACS Brief particularly recommended for screening in at-risk populations (e.g., after severe head trauma or in psychosis). Another limitation to consider is the fact that APACS Brief returns a single coarse-grained score of pragmatic skills, without additional fine-grained task scores as in APACS. Thus, if subtle weaknesses in pragmatic skills are being assessed and/or a more comprehensive pragmatic profile is needed, for example, to guide personalized rehabilitative intervention, the full APACS test or some of its tasks may be more appropriate.

At this point, it is worth highlighting that, while considering both expressive and receptive facets of pragmatic competence, APACS Brief items test a specific selection of pragmatic phenomena, focusing on discourse production and the understanding of non-literal language. For a broader picture of pragmatic profiles (e.g., see Frau et al., 2024b), aspects such as speech acts and irony comprehension could be integrated to extend the assessment scope. There is a variety of ad hoc tasks that could be adapted for clinical groups, from recognizing ironic remarks (Bettelli et al., 2024; Pfeifer & Pexman, 2024) to creating effective referential expressions or rating the function, coherence, directness, and predictability of direct and indirect replies (Arvidsson et al., 2022; Boux et al., 2023), as well as judging meanings conveyed via different politeness strategies (Gotzner & Scontras, 2024; Mazzarella et al., 2018). Increasing the assessment scope could be useful to capture nuances of individual differences in pragmatic competence, which are known to vary along multiple dimensions (Frau et al., 2024a; Honan et al., 2015; Surian, 1996; Varga et al., 2013).

Related to the utility of using brief versions for over-time monitoring or to reduce practice, memory, and carry-over effects, APACS Brief comes with an Alternate Form that, we showed, has equally good psychometric properties compared to the main version. In particular, the two versions were found to be moderately correlated, not significantly different and equal to each other, with total scores describing in a similar manner the overall pragmatic competence of neurotypical adults. The moderate correlation was expected, since the two versions (the Main and the Alternate forms) included different items, and is in line with the magnitude of the association between main and alternate forms of other tests (see, e.g., *r* = 0.44 reported by Saa et al., 2017). The equivalence testing further supports the discussion of these forms as alternate (Furr & Bacharach, 2008). The advantages of APACS Brief extend also to the positive experience reported by participants in the debriefing. APACS Brief was evaluated as not a burdensome test, which suggests that this tool can help manage not only time but also the physical and mental resources of patients. A minor point remains to be discussed, namely the (small) practice effect reported for the main form of the APACS Brief test. In light of the availability of two forms, this practice effect can be considered of little importance, since the Alternate Form can be profitably used for post-training or follow-up evaluations in the context of intervention programs promoting pragmatic skills (e.g., Bambini et al., 2022, 2020c; Bosco et al., 2016; Gabbatore et al., 2015; Jagoe, 2020).

Our work also offers interesting insights into pragmatic skills from a lifetime perspective. Besides further supporting the validity of APACS Brief, the regression analysis detailed the extent to which demographic features account for the variability in pragmatic competence, resulting in age- and education-related effects. Age was negatively associated with APACS Brief scores: the decline of pragmatic skills in the golden age has been largely described (Baraldi & Domaneschi, 2024; Bischetti et al., 2023; Ceccato et al., 2025; Mazzaggio et al., 2023). Here we showed that this trend follows a curvilinear pattern: pragmatic skills continue growing beyond late adolescence and early adulthood (Fussman & Mashal, 2022), peaking between 30 and 45 years of age (as sketched in the pioneering work by Nippold et al., 1997), adding to asynchronous rise and fall of several aspects of cognition through the lifespan (Hartshorne & Germine, 2015). APACS Brief scores were positively associated—on a linear scale—with attained education, as well as with the Education index of the CRIq (which largely describes educational and academic achievements): this correlation further confirms the validity of our results, and it is in line with previous findings relating attained education to pragmatic skills (Angeleri et al., 2012; Arcara & Bambini, 2016; Petit et al., 2025; Sacco et al., 2008) and to cognitive skills in general (Strauss et al., 2006). In addition, our data confirm that sex plays a negligible role in global pragmatic skills, with its effect possibly becoming relevant only in specific cases (e.g., some types of caustic jocularity or dark humor, see Bischetti et al., 2021; Ivanko et al., 2004). Importantly, the cut-off scores provided with APACS Brief were calculated taking into account relevant age and education aspects.

To sum up, this study demonstrates the feasibility of assessing pragmatic skills within a short timeframe, providing a reliable and valid tool in Italian with satisfactory psychometric properties, enriching the current panorama of tests in the *APACS family* (APACS, APACS Brief and APACS Brief Remote, with already available translations in Flemish, Hebrew, and French and ongoing adaptations to other languages; Bambini et al., 2021; Fussman & Mashal, 2022; Petit et al., 2025). This could encourage the future use of this test in various contexts, supporting the integration of pragmatics into clinical routine assessment and the evaluation of linguistic and communicative abilities in other contexts. Finally, in the spirit of Open Science, the APACS Brief test, manual, scoring sheets, and cut-off scores are freely distributed under a Creative Commons license to further promote its adoption by interested professionals in clinical or research settings.

## Figures and Tables

**Table 1:** Results of APACS Brief and cognitive assessment.

	Mean	SD	Median	Min	Max	Kurtosis	Skewness	Q1	Q3
Age	43.94	17.04	43	18	81	1.77	0.22	27	57.5
Education	14.01	3.81	13	5	24	2.43	−0.07	13	17
APACS Brief Total [0–1]	0.84	0.11	0.87	0.35	1	4.78	−1.05	0.77	0.93
Interview [0–4]	3.76	0.49	4	2	4	5.83	−1.91	4	4
Narratives [0–6]	4.70	0.97	5	1	6	3.11	−0.57	4	5
Figurative Language 1 [0–3]	2.89	0.36	3	0	3	20.55	−3.71	3	3
Humor [0–2]	1.78	0.44	2	0	2	4.89	−1.72	2	2
Figurative Language 2 [0–3]	1.90	0.93	2	0	3	2.22	−0.39	2	3
Interview (prop.) [0–1]	0.94	0.11	1	0.50	1	5.83	−1.91	1	1
Narratives (prop.) [0–1]	0.78	0.16	0.83	0.17	1	3.11	−0.57	0.67	0.83
Figurative Language 1 (prop.) [0–1]	0.96	0.12	1	0	1	20.55	−3.71	1	1
Humor (prop.) [0–1]	0.89	0.22	1	0	1	4.89	−1.72	1	1
Figurative Language 2 (prop.) [0–1]	0.63	0.31	0.67	0	1	2.22	−0.39	0.33	1
APACS Total [0–1]	0.94	0.04	0.94	0.81	1	4.22	−1.12	0.92	0.96
APACS Brief Remote Total [0–1]	0.86	0.11	0.90	0.63	1	2.69	−0.62	0.80	0.93
WAIS-R Vocabulary subtest (raw score) [0–70]	50.73	9.63	51	25	69	2.44	−0.30	44	58
GEMS (adjusted score) [0–100]	83.36	8.02	84.2	47.95	95	6.07	−1.48	80.23	89.15
TIB (Total IQ)	109.60	5.52	110.16	90.50	121.16	3.22	−0.57	106.45	113.62
Verbal IQ	109.28	5.45	109.90	89.63	120.41	3.48	−0.67	106.20	112.85
Performance IQ	109.37	5.66	109.58	92.79	120.58	2.69	−0.23	105.62	113.54
CRIq	112.91	20.05	110	74	165	2.56	0.57	97	126
CRI school	105.15	13.48	104	75	154	3.64	0.69	96	112.5
CRI work	103.71	12.68	101	72	147	2.95	0.54	93.5	1140
CRI leisure time	120.14	28.26	112	72	194	2.53	0.77	97	138.5

Note. Values for the APACS Brief total score and task scores (also in proportional (prop.) scores) from the whole sample of participants who completed the assessment sessions (n = 287) are reported. Ranges of possible scores for each test or task—when available—are reported in square brackets. Min and Max depict the range of observed scores. Kurtosis was estimated with Pearson’s measure, and normally distributed data should have kurtosis = 3. Age and education are reported in years. APACS data were available for a sample of 73 neurotypical adults (Age: M = 42.14, SD = 13.01; Education: M = 14.80 years, SD = 4.06; 50F). APACS Brief Remote data were available for a sample of 21 neurotypical adults (Age: M = 40.29, SD = 13.12; Education: M = 16.00 years, SD = 2.59; 11F). Wechsler Adult Intelligence Scale–Revised (WAIS-R) vocabulary subtest data were available for a sample of 152 adults (Age: M = 43.23 years, SD = 15.97; Education: M = 13.30 years, SD = 3.78; 99F); following the administration procedure and the stopping rule, we post-hoc removed subjects scoring below the 5th percentile (n = 8). Global Examination of Mental State (GEMS) data were available for a sample of 163 adults (Age: M = 45.51 years, SD = 18.28; Education: M = 14.45 years, SD = 4.06; 99F). Test di Intelligenza Breve (TIB) data were available for a sample of 166 adults (Age: M = 45.92 years, SD = 18.40; Education: M = 14.39 years, SD = 4.06; 101F). Cognitive reserve (CRIq) data were available for a sample of 151 adults (Age: M = 46.79 years, SD = 18.89; Education: M = 14.20 years, SD = 4.09; 91F). The slight variability in sample numerosity across assessment measures originates from the concatenation of data points across the study arms, and from occasional circumstances where participants were unable to take part in the full assessment but were retained for the analysis since they completed—at least—the APACS Brief test once.

## Data Availability

The data generated and analyzed during the current study are available in the OSF repository, https://osf.io/cenx8/, (accessed on 1 January 2025)). The APACS Brief test will be distributed freely under a CC BY-NC-ND 4.0 license. It will be possible to obtain it from the authors or by accessing the NEPLab website (https://www.neplab.it/apacs/, (accessed on 1 January 2025)). The NEPLab website will also host a software application for rapid and easy scoring.

## Note

1 In running this set of analyses, we removed two subjects, each of whom represented a single observation for Education (i.e., 23 and 24 years) and biased the computation of estimates. The final models were run on 285 for the APACS Brief and 79 participants for the APACS Brief Alternate form.
